# Contrasting relapse rate associated with the absence of HLA-A3/A11 in T cell-replete haploidentical transplantation

**DOI:** 10.3389/fimmu.2026.1907346

**Published:** 2026-08-25

**Authors:** Xiang-Feng Tang, Hai-Fei Zhou, Wei-Jie Zhang, Yan-Hui Luo, Xiao-Qi Wang, Xing-Yu Cao, Xiao-Dong Wang, Bin Wang, Yi-Mei Feng, Guang-Hua Zhu, Chen-Guang Jia, Ying-Jian Si, Wei Lu, Mao-Quan Qin, Xiang-Jun Liu, Jing-Bo Wang

**Affiliations:** 1 National Engineering Laboratory for Birth Defects Prevention and Control of Key Technology, Beijing Key Laboratory of Pediatric Organ Failure, Department of Pediatrics, The Seventh Medical Center of PLA General Hospital, Beijing, China; 2 Beijing BFR Gene Diagnostics, Beijing, China; 3 Department of Hematology, Aerospace Center Hospital, Beijing, China; 4 Department of Hematology and Oncology, Beijing Children’s Hospital, Capital Medical University, Beijing, China; 5 Medical Centre of Haematology, Xinqiao Hospital of Army Medical University, Chongqing, China; 6 Department of Bone Marrow Transplant, Hebei Yanda Lu Daopei Hospital, Langfang, China; 7 Blood and Marrow Transplantation Center, Shanghai Children’s Medical Center, Shanghai Jiao Tong University School of Medicine, Shanghai, China

**Keywords:** NK cell education, alloreactivity, haploidentical transplantation, killer immunoglobulin-like receptor, relapse

## Abstract

**Introduction:**

Natural killer cell alloreactivity contributes to the graft-versus-leukemia (GVL) effect of haploidentical hematopoietic stem cell transplantation (haplo-HSCT). However, its importance remains controversial across studies.

**Methods:**

In this retrospective analysis, we studied NK cell alloreactivity and its association with clinical outcomes using the education model in patients with hematologic malignancies undergoing transplantation. Patients were stratified by the presence or absence of alloreactivity assessment, which was defined by the coexistence (in the donor) of four germline-encoded inhibitory killer cell immunoglobulin-like receptors (iKIRs) and their corresponding self-human leukocyte antigen (HLA)-I ligands.

**Results:**

In a cohort of 1,209 patients,793 individuals (65.6%) had no loss of iKIR ligands, while 416 cases showed alloreactivity characterized by the absence of at least one ligand. Among the missing single ligand, the absence of HLA-A3/A11 epitope was the most prevalent (28.4%), followed by Bw4 (25.2%), C2 (23.6%), and C1 (10.6%). Only patients with KIR3DL2-A3/A11-mediated alloreactivity exhibited a substantially reduced 3-year cumulative incidence of relapse (CIR) compared to those without this predicted alloreactivity (16.2%; 95% confidence interval [CI], 10.1-23.5%; and 30.1%; 95% CI, 27.1-33.3%; *P* = 0.0006). Multivariate analysis confirmed A3/A11 deficiency as an independent protective factor against relapse (adjusted hazard ratio 0.487; 95% CI, 0.300-0.790; *P* = 0.0035). Moreover, alternative predictive models failed to reliably forecast relapse risk.

**Discussion:**

Our result suggest that NK cell alloreactivity driven by the absence of HLA-A3/A11 may enhance NK cell-mediated surveillance against leukemia cells; therefore, incorporating KIR3DL2-A3/A11 compatibility assessment can refine donor selection strategies aimed at reducing relapse incidence in patients undergoing haplo-HSCT.

## Introduction

Haploidentical hematopoietic stem cell transplantation (haplo-HSCT) represents an effective therapeutic option for patients with hematological malignancies who lack an HLA-matched donor. Despite its clinical efficacy, relapse remains the leading cause of mortality following haplo-HSCT (1, 2). Natural killer (NK) cells serve as critical early mediators of anti-tumor immunity and offer the compelling advantage of potent graft-versus-leukemia (GVL) activity without exacerbating graft-versus-host disease (GVHD) (2, 3). NK cells express clonally distributed inhibitory receptors known as killer cell immunoglobulin-like receptors (KIRs), which specifically recognize self-human leukocyte antigen (HLA) class I molecules (4, 5). Donor NK cell alloreactivity depends on their capability to detect the absence of cognate self-HLA ligands in the recipient (6–9). A few retrospective analyses of large haplo-HSCT cohorts have associated predicted NK alloreactivity with reduced leukemia relapse rates or improved survival (6–12). However, other studies have failed to replicate these associations (7, 13–16). To date, genetic algorithms incorporating donor-recipient HLA and KIR genotypes to predict NK cell alloreactivity have yielded inconsistent results, thereby precluding definitive clinical implementation. The inherent complexity of HSCT further complicates the identification of conditions that either promote or suppress NK cell-mediated alloreactivity.

The education model for predicting NK cell alloreactivity posits that donor NK cells become functionally competent (“licensed”) through interactions between their inhibitory KIRs and self-HLA class I ligands in the donor (9, 17–20). When these licensed NK cells encounter a recipient who lacks the corresponding HLA ligand, they are predicted to mediate alloreactivity against recipient cells, including leukemia cells (GVL effect) (21). In the studies mentioned above, when analyzing KIRs and theirs ligands, the three most common epitopes, namely C1, C2, and Bw4, are generally taken into consideration; whereas A3 or A11 along with their receptor KIR3DL2 are used less frequently in such analyses (2, 6, 12). In a pilot study, we utilized the education model to assess the clinical impact of KIR3DL2-A3/A11-mediated alloreactivity and found its association with lower relapse risk following haplo-HSCT (17). However, the finding was constrained by a limited sample sizes, leaving unresolved key questions regarding the magnitude of the effect and statistical robustness of this effect as an independent factor prognostic factor. In this study, we therefore leveraged a large, multiple-center cohort to rigorously evaluate the impact of NK cell alloreactivity on clinical outcomes. We hypothesize that the absence of the HLA-A3/A11 in recipients, when paired with donors harboring licensed KIR3DL2-positive NK cells, may serve as an independent biomarker for predicting relapse following T cell-replete haploidentical transplantation.

## Materials and methods

### Study design and cohort

This was a multiple-center, retrospective cohort study designed to evaluate the association between NK cell alloreactivity, as predicted by the education model, and clinical outcomes. We analyzed 1209 patients diagnosed with acute myeloid leukemia (AML), acute lymphoblastic leukemia (ALL), or other malignant hematologic disorders who received their first haplo-HSCT between 2013 and 2023 as per Beijing protocol as described (23–25). The analysis was approved by the institutional ethics committee (S2025-146-01), and informed consent was obtained in accordance with the Declaration of Helsinki.

### NK cell licensing systems and alloreactivity assessment

We specifically assessed the licensing status of four principal inhibitory KIR-ligand pairs based on the education model: KIR2DL2/3 with C1, KIR2DL1with C2, KIR3DL1 with Bw4, KIR3DL2 with A3/A11. NK cell alloreactivity was defined by the coexistence (in the donor) of germline-encoded iKIRs and their corresponding self-HLA-I ligand: when a donor carries the cognate HLA ligand for a given iKIR, their NK cells undergo self-licensing and remain tolerance toward autologous cells; conversely, if the recipient lacks that specific ligand, donor-derived NK cells, which were uninhibited by missing self-recognition, become activated and mediate lysis of recipient target cells in the allogeneic setting. Patients were stratified into two groups according to the licensing status: those with alloreactivity if they lacked at least one of these licensing ligands present in their respective donors (ALLO group), and those without non-alloreactivity if they possessed the corresponding ligand (Non-ALLO) group. Donors lacking either the relevant KIR or its cognate ligand were deemed non-alloreactivity. Theoretically, alloreactive NK cells may be missing up to four ligands, or none, depending on donor KIR and HLA genotype.

### KIR and HLA typing

Cryopreserved specimens were used for DNA extraction and supplementary KIR genotyping. The presence or absence of various KIR genes (*KIR2DL1, KIR2DL2/3, KIR2DL4, KIR2DL5A, KIR2DL5B, KIR2DS1, KIR2DS2, KIR2DS3, KIR2DS4, KIR2DS5, KIR3DL1/3DS1, KIR3DL2, KIR3DL3*) and two KIR pseudogenes (*KIR2DP1* and *KIR3DP1*) was determined for all donors and patients utilizing a commercially available KIR-SSO kit (Immucor GTI Diagnostics, Waukesha, USA) following the manufacturer’s instructions. Amplicon quantification was carried out on the Luminex LABScanTM 100 flow analyzer. HLA genotyping at five loci (HLA-A, -B, -C, -DRB1, and -DQB1) was performed via sequencing-based typing (SBT) with GenDx kits (Utrecht, Netherlands).

### Endpoints and statistical analysis

The primary endpoint was relapse, and its cumulative incidences was analyzed. The presence of 5% or more leukemic cells in the bone marrow and no indication of extramedullary localization was considered a hematological relapse. The cumulative incidence of relapse (CIR) was estimated utilizing the Fine and Gray subdistribution hazard model, considering non-relapse mortality (NRM) as a competing risk.Overall survival (OS) were depicted using Kaplan-Meier curves stratified by predicted alloreactivity-defined groups. Inter-group CIR and OS comparisons were performed using the Gray test and the log-rank test, respectively. Continuous variables were reported as medians with interquartile range (IQR) and were compared using the Mann-Whitney U test. Categorical variables were expressed as frequencies (percentages) and were compared using the χ² or Fisher’s exact test, as appropriate. Comparisons among multiple groups were performed using the Kruskal-Wallis test.

Multivariate analyses were performed for OS using the Cox proportional hazards regression model, and CIR using the Fine and Gray proportional subdistribution hazards regression model. The analyses were adjusted for pre-specified clinical covariates including disease status at transplant (NR *vs*. CR), disease type, donor age, and HLA compatibility between the donor-recipient pair. Effect sizes were reported as adjusted hazard ratios (aHR) with their corresponding 95% confidence intervals (95% CI).

All statistical tests were two-sided, with significant threshold set at P < 0.05. Follow-up duration for all outcomes exceeded three months. Patients without events were censored at the date of their last clinical assessment. Statistical analyses were conducted using the graphical user interface for R software, EZR version 1.70 (26).

## Results

### Population characteristics

A total of 1209 recipient-donor pairs were included, of which 186 were derived from a small cohort study published in 2025 (17). Employing the education model, 416 patients (34.4%) were predicted to have NK cell alloreactivity (ALLO group). The remaining 793 patients (65.6%) constituted the Non-ALLO group. The median age at transplantation was 16.0 years (IQR: 8.0-31.0) in the Non-ALLO group, 14.0 [6.3-30.0] in the ALLO-missing licensing C1/C2/Bw4 group and 20.0[9.0, 33.0] in the ALLO-missing licensing A3/A11 group. Among the 565 adults in the cohort, 143 experienced relapse (25.3%), 114 died from non-relapse causes (NRM; 20.2%), and 308 remained no relapse or death. Among the 644 pediatric patients, 167 relapsed (25.9%), 70 died from NRM (10.9%), and 407 remained event-free (no relapse or death). The vast majority of patients were diagnosed as AML/MDS and ALL, collectively accounting for >97% of cases across both ALLO and Non-ALLO groups. The baseline characteristics of the three cohorts are summarized in **Table 1**.

**Table 1:** Population characteristics.

Characteristic	Non-ALLO group	ALLO group		*P*
		C1/C2/Bw4 missing	A3/A11 missing	
Number of patients	793	270	146	
Patients age-year [median (IQR)]	16.0 [8.0, 31.0]	14.0 [6.3, 30.0]	20.0 [9.0, 33.0]	0.067
Child-n (%)	416 (52.5)	164 (60.7)	64 (43.8)	
Adult-n (%)	377 (47.5)	106 (39.3)	82 (56.2)	
Disease status at transplant-n (%)				0.400
CR/PR	633 (79.8)	213 (78.9)	124 (84.9)	
NR	161 (20.2)	57 (21.1)	22 (15.1)	
Disease diagnosis-n (%)				0.507
ALL	413 (52.1)	128 (47.4)	63 (43.1)	
AML/MDS	372 (46.9)	136 (50.4)	79 (54.1)	
JMML	3 (0.4)	4 (1.5)	2 (1.4)	
MPAL	3 (0.4)	2 (0.7)	0	
others*	2 (0.3)	0	2 (1.4)	
TBI-n (%)				0.400
NO	527 (72.9)	210 (77.8)	118 (80.8)	
YES	196 (27.1)	60 (22.2)	28 (19.2)	
Donor KIR haplotype-n (%)				0.067
AA	388 (48.9)	147 (54.4)	78 (53.4)	
Bx	405 (51.1)	123 (45.6)	68 (46.6)	
B content score-n (%)				0.342
0	391 (49.3)	147 (54.4)	78 (53.4)	
1	248 (31.3)	85 (31.5)	48 (32.9)	
2	120 (15.1)	35 (13.0)	13 (8.9)	
3	30 (3.8)	3 (1.1)	6 (4.1)	
4	4 (0.5)	0	1 (0.7)	
ct-KIR-n (%)				0.196
0	197 (24.8)	80 (29.6)	36 (24.7)	
1	513 (64.7)	172 (63.7)	97 (66.4)	
2	83 (10.5)	18 (6.7)	13 (8.9)	
HLA match-n (%)				0.065
5/10	385 (48.5)	181 (67.1)	98 (67.1)	
6/10	172 (21.7)	49 (18.1)	17 (11.6)	
7/10	113 (14.2)	31 (11.5)	26 (17.8)	
8/10	61 (7.7)	6 (2.2)	3 (2.1)	
9/10	53 (6.7)	2 (0.7)	2 (1.4)	
10/10	9 (1.1)	1 (0.4)	0	

ALL, acute lymphocytic leukemia; AML, acute myelocytic leukemia; MDS, myelodysplastic syndromes; JMML, juvenile myelomonocytic leukemia; MPAL, mixed phenotype acute leukemia; TBI, total body irradiation; CR, complete remission; NR, non-remission.*Others included: acute non-lymphocytic leukemia; T-lymphoblastic lymphoma; myeloid sarcoma.

### Distribution of NK cell licensing system based on the education model

**Figure 1:**
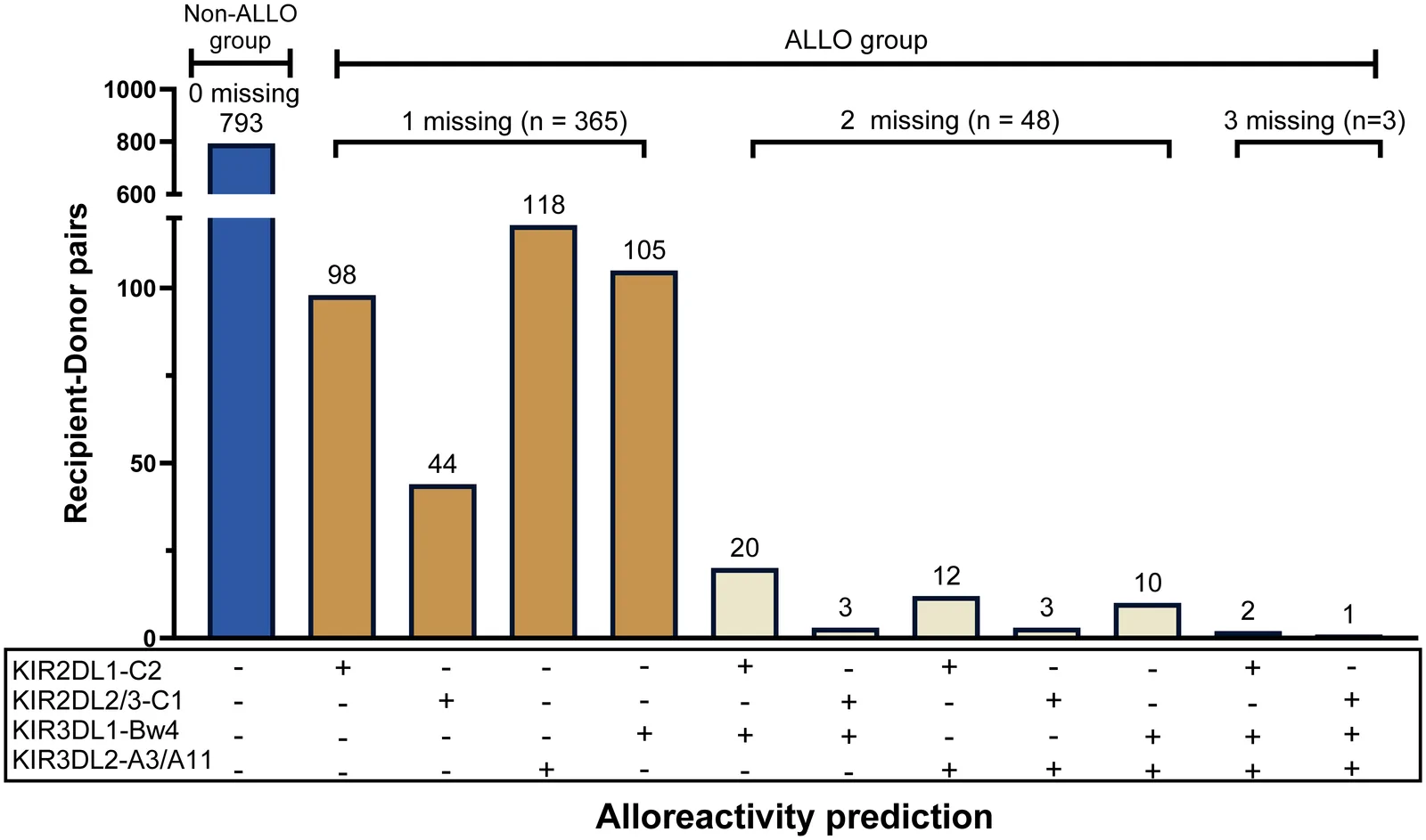
Characterization and distribution of predicted alloreactivity based on missing licensing according to the education model. The ALLO cohort (n = 416) is defined by ≥1 missing ligand among the four inhibitory KIR-HLA licensing pairs: KIR2DL1-C2, KIR2DL2/3-C1, KIR3DL1-Bw4, and KIR3DL2-A3/A11. Among 365 patients exhibiting a single missing ligand, the absence of A3/A11 is the most prevalent (n = 118, 32.3%), followed by Bw4 (n = 105, 28.8%), C2 (n = 98, 26.8%), and C1 (n = 44, 12.1%). Within the subset of 48 patients with two missing ligands, the combination of A3/A11 and C2 is the most frequent (n = 20, 41.7%). Three patients demonstrate the absence of three ligands, all involving A3/A11 and Bw4 in conjunction with either C1 or C2.

Among the ALLO group, patients lacking exactly one licensing ligand constituted the largest subset (n = 365, 87.7%), followed by those lacking two ligands (n = 48, 11.5%) (**Figure 1**). Patients lacking more than two licensing ligands were rare: only 0.72% (n = 3) lacked three of the four assessable canonical ligands, and no patient was found to lack all four simultaneously. The most frequent single missing ligand was HLA-A3/A11, accounting for 28.4% (n = 118) of ALLO cases. The distribution of other single-ligand missing patterns was: Bw4 (25.2%, n = 105), C2 (23.6%, n = 98), and C1 (10.6%, n = 44) (**Figure 1**). The most common dual-ligand deficiency was Bw4 + C2 (n = 20, 4.8%), followed by A3/A11 + C2 (n = 12, 2.9%), and Bw4 + A3/A11 (n = 10, 2.4%) (**Figure 1**). Three patients lacked three ligands: two cases involved the absence of Bw4, A3/A11 and C2, while one case involved the absence of Bw4, A3/A11 and C1 (**Figure 1**). Overall, 146 patients had a missing A3/A11 ligand, either alone or in combination with the absence of other ligands.

### Impact of NK cell alloreactivity on clinical outcomes

**Figure 2:**
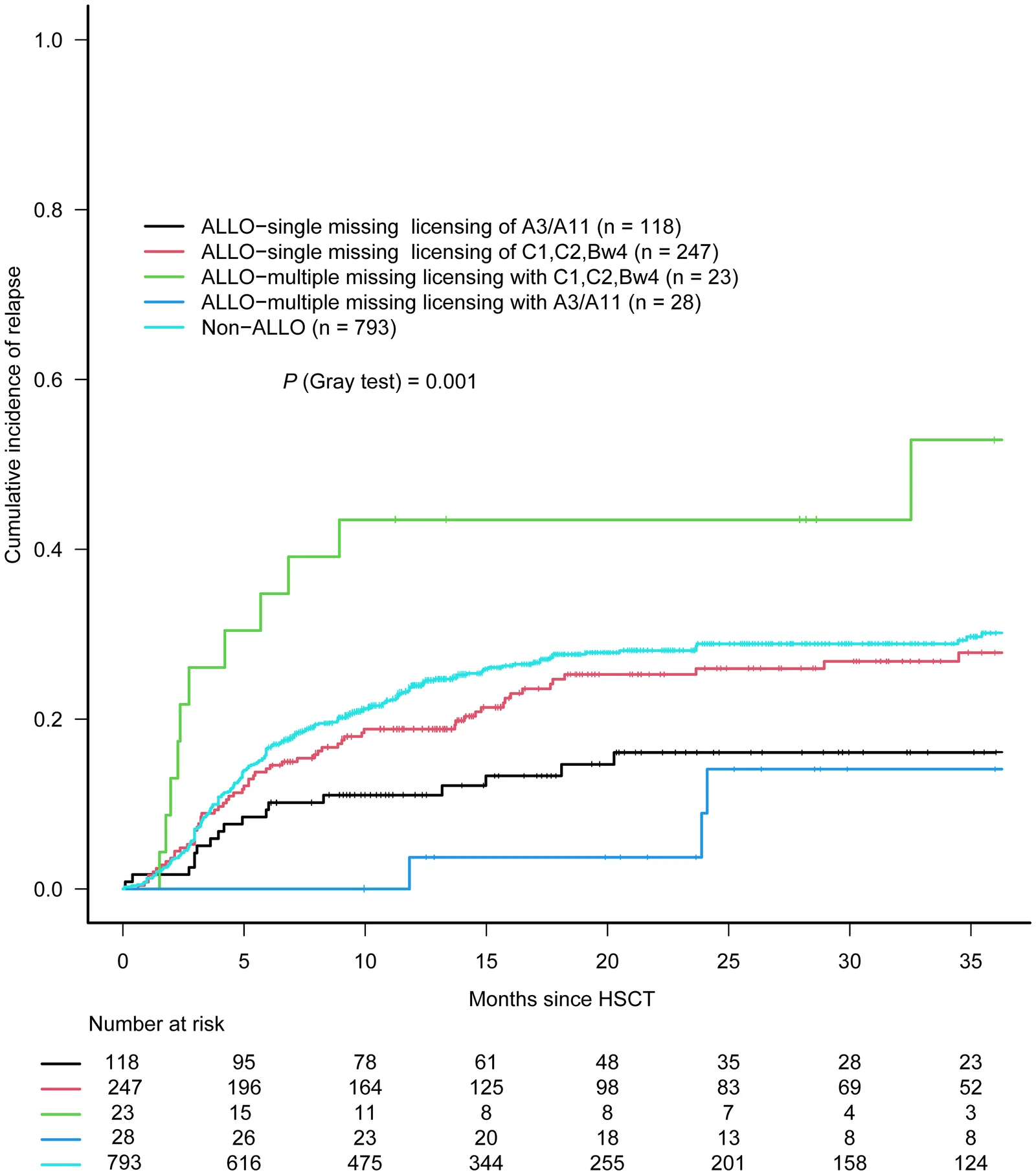
Cumulative incidence curve for relapse in patients with the presence or absence of predicted alloreactivity according to the education model. ALLO group with only missing A3/A11 (n = 118) exhibited significantly a lower 3-year CIR compared to the Non-ALLO group (n = 793) (16.1% vs. 30.1%; *P* = 0.0058). No significant difference in 3-year CIR was observed among patients with predicted alloreactivity defined by single missing C2, C1, or Bw4 (n = 247). The ALLO-multiple missing licensing with A3/A11 group (n = 28) exhibited a significantly low CIR compared to the ALLO-multiple missing licensing with C1/C2/Bw4 group (n = 23; *P* = 0.0023). Statistical analyses were conducted using Gray’s test.

The 3-year CIR for the entire Non-ALLO group was 30.1% (95% CI, 26.6-33.8%). Patients lacking any one of Bw4, C2, and C1 showed 3-year CIRs of 27.0% (95% CI, 17.5-37.3%), 29.2% (19.4-39.7%), and 28.9% (15.9-43.3%), respectively. The 3-year CIR for these patients was comparable to the Non-ALLO group, with no statistically significant differences observed (all *P* > 0.05). Patients lacking only A3/A11 licensing (n = 118) had a significantly lower 3-year CIR of 16.1% (95% CI, 9.6-23.9%), compared to 30.1% in the Non-ALLO group (**Figure 2**; *P* = 0.0058), implying that NK cells from donors licensed via KIR3DL2-A3/A11 can mount an effective GVL effect against leukemia cells in A3/A11-deficient recipients. Fifty-one recipients with multiple missing licensing ligands were stratified into 2 groups: one group related to A3/A11 (n = 28), and the other involved C1, C2 and Bw4 (n = 23). As illustrated in **Figure 2**, ALLO-multiple missing licensing with A3/A11 group also displayed a significantly lower 3-year CIR of 14.1% (95% CI, 3.2-32.7%) relative to the Non-ALLO group (CIR, 30.1%; 95% CI, 26.6-33.8%; *P* = 0.0278) and ALLO-multiple missing licensing with C1, C2 and Bw4 group (CIR, 52.9%; 95% CI, 25.5-74.3%; *P* = 0.0228). Specifically, when the loss of licensing A3/A11 was compounded by the absence of additional licenses, the transplanted NK cells exhibited enhanced higher anti-tumor activity, thereby reducing the risk of relapse in recipients of haploidentical transplantation. Together, analysis of specific ligand-missing patterns revealed a significant protective effect associated with the absence of the HLA-A3/A11 ligand.

**Figure 3:**
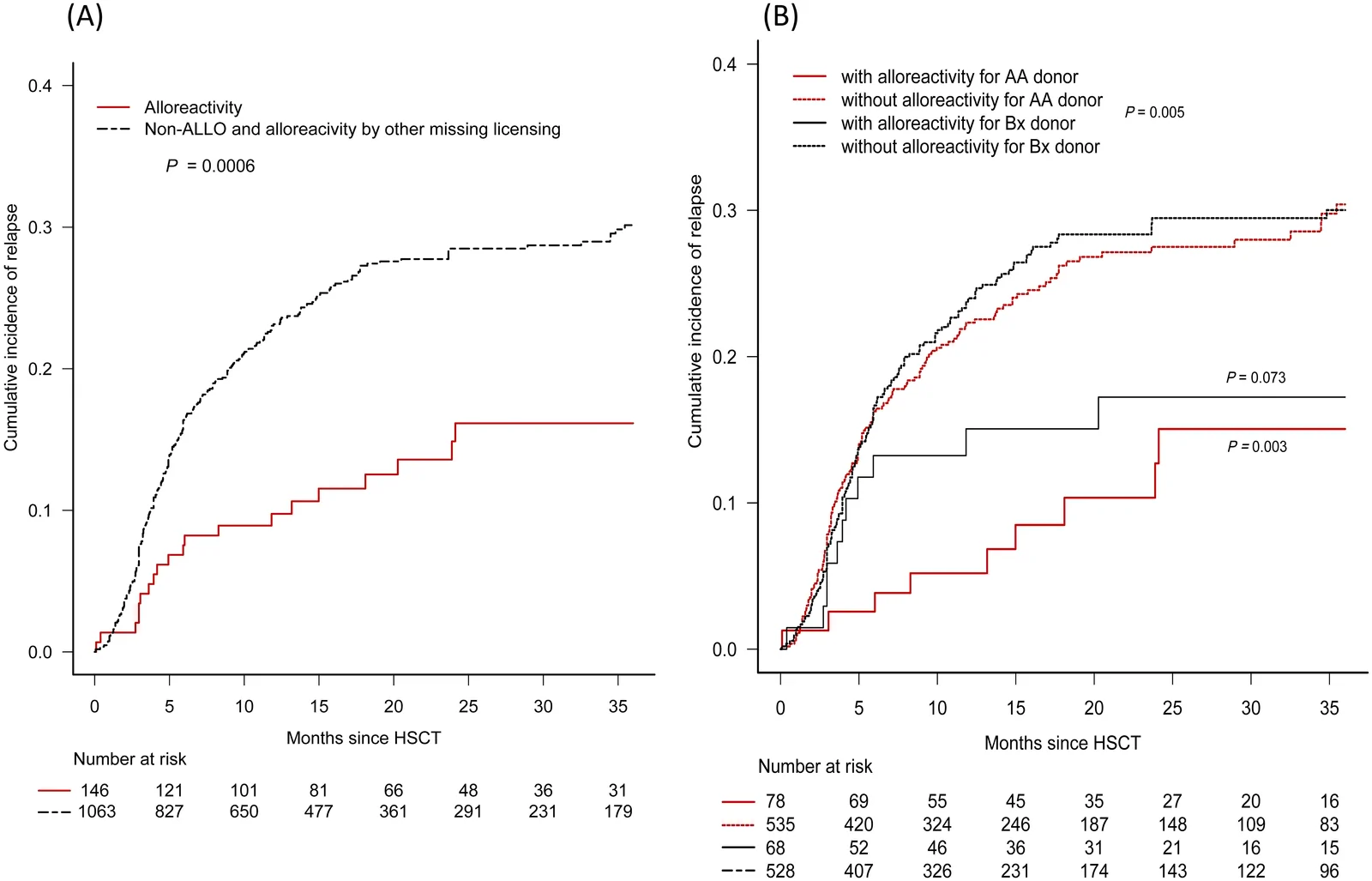
Comparison of cumulative incidence of relapse within the whole cohort **(A)** and within subgroups **(B)**.

The protective effect was consistent when analyzing all patients with missing A3/A11 ligand (n = 146, includes those with combined deficiencies), showing a 3-year CIR of 16.2% (95% CI, 10.1-23.5%), which remained significantly lower than other cases (30.1%; 95% CI, 27.1-33.3%) (**Figure 3a**; *P* = 0.0006). Moreover, no specific P-value for OS comparison stratified solely by A3/A11 status was extractable from the dataset. For AA donors, the relapse rate with alloreactivity mediated by the KIR3DL2-A3/A11 axis was significantly lower than those without alloreactivity (**Figure 3b**; *P* = 0.003). A similar trend was observed for Bx donors, but did not reach statistical significance (**Figure 3c**; *P* = 0.073). Concurrently, the relapse rate with alloreactive AA donors was lower than that with alloreactive Bx donors, in line with the previous report that identified a protective effect from developing leukemia or lymphoma for AA carriers in Chinese population (22, 23). We further evaluated the effects of the activating receptors KIR2DS2 and KIR2DS4, which are thought to bind with ligand A11. The results indicated that their binding with A11 did not have a significant impact on the relapse rate or overall survival. The overall analysis indicated that the clinical benefit was primarily driven by reduced relapse risk. To contextualize our findings, we evaluated the predictive power of several established models (Haplotype model, B-content model, ct-KIR score, CF-iKIR score) in our cohort. As a result, these traditional models did not have a significant correlation with relapse and overall survival, highlighting the unique and dominant role of the KIR3DL2-A3/A11 axis under the specific immunological conditions of T cell-replete haplo-HSCT using the Beijing protocol. Taken together, these findings suggest that loss of the licensing A3/A11 enhances NK cell-mediated tumor surveillance and may protect against disease relapse; however, this effect shows no significant association with the absence of C1, C2 and/or Bw4.

Multivariate analysis revealed that the missing A3/A11 ligand was independent protective factor against relapse. The adjusted hazard ratio (aHR) was 0.487 (95% CI, 0.300-0.790; *P* = 0.0035). This finding indicates that, after adjustment for other significant clinical covariates, the presence of this specific NK cell alloreactivity potential was associated with a statistically significant ~51% reduction in relapse risk. Disease status at transplant and donor age showed another independent risk factors for relapse risk, with a HR of 3.185 (95% CI, 2.382-4.259; *P* < 0.0001) and 0.987 (95% CI, 0.978-0.995; *P* = 0.0023). While KIR3DL2-A3/A11 incompatibility is a significant protective factor, multivariate analysis confirmed that the disease status at transplantation remains the most powerful predictor of relapse. Patients transplanted in non-remission faced a substantially higher risk of relapse compared to those in complete remission. This underscores that while donor selection based on KIR-HLA can improve outcomes, achieving disease control prior to transplant is paramount.

## Discussion

Our study demonstrates, in a large, ethnically homogeneous cohort of patients undergoing T cell-replete haplo-HSCT, that NK cell alloreactivity mediated by the missing HLA-A3/A11 is an independent and potent protective factor against relapse. An approximate 51% reduction in relapse risk highlights its substantial clinical effect size. Conversely, the absence of other canonical licensing ligands or ligand mismatches showed no significant benefit, and established predictive models had no prognostic value as well. These findings compel a re-evaluation of the dominant framework for predicting NK-mediated GVL effects in the context of contemporary haplo-HSCT.

While KIR3DL2 is expressed on a substantial subset of NK cells, its role in the process of NK cell “education” or “licensing” remains a subject of ongoing debate. *In vitro* studies suggest that NK cells expressing only KIR3DL2 may remain hyporesponsive even in the presence of its ligand A3 or A11, potentially requiring co-expression of other receptors or cytokine priming for full functional maturation (27). KIR3DL2-single positive NK cells has been founded to coexpress NKG2A more frequently than NK cells expressing any of the other three iKIR (27). Intriguingly, this coexpression can partially compensate for impaired education observed in KIR3DL2-single positive NK cells. Nevertheless, *in vitro* assays fail to fully recapitulate the complex and dynamic nature of human NK cell education observed *in vivo*. Within the complex and dynamic immune reconstitution milieu post-transplant, which is shaped by immunosuppression, G-CSF administration, and inflammatory cytokines, the functional licensing of KIR3DL2^+^ NK cells may be dynamically “reset”. This phenomenon of “downward reset” or “upward reset” of the licensing has been observed in adoptive transfer of NK cells (28, 29). It seems that the protective effect is not attributed to a direct cytolytic activity, but rather to “re-licensing” during or after the process of protocol-specific immune reconstitution.

Another possible explanation for the revealed association with KIR3DL2-A3/A11 axis might be the profound impact of transplant platform specifics (12, 24, 30). Specifically, T-cell-depleted protocol used an average infusion of 3×10^6^ T cells/kg, whereas Beijing protocol employed stem cell inocula containing a substantially higher median T cell dose of 1.5 × 10^8^ T cells. Protocols employing higher doses of T cells may induce more intense T-cell-mediated alloreactivity, which could mask or negate any beneficial GVL effect contributed by NK cells. Moreover, *in vivo* application of G-CSF for donor stem cell preparation leads to an increased ratio of T and NK cells and decreased ratio of CD56^dim^ and CD56^bri^ NK cells, ultimately decreasing the cytotoxicity of NK cells (25). Thus, focusing solely on NK cell cytotoxicity while overlooking their immunomodulatory functions in the context of haplo-HSCT fails to fully elucidate the underlying mechanisms. Established models focused predominantly on the C1/C2/Bw4 triad, potentially overlooking the contribution of A3/A11. Our finding suggest that this omission may lead to an incomplete and sometimes contradictory assessment of “alloreactive” potential.

Finally, differences in the frequencies of specific HLA class I ligands in various populations might explain the differing results. Deng et al. identified a protective effect from developing hematologic malignancies for KIR AA haplotype in Chinese Han population, while large case-control study showed no effect on the susceptibility for the development of *de novo* AML in European populations (31, 32). The team also revealed a greater abundance of HLA-A and -B ligands in East Asians than other populations as well as a greater diversity of interactions between inhibitory KIR and HLA-A and -B (33). Specially, the frequency of HLA-A11 in Chinese Han population, representing the world’s largest population group, is significantly higher than that in European or other populations (33, 34). Our results seemed to verify Deng’s hypothesis that NK cells were efficiently educated in KIR AA haplotype-bearing individuals to detect loss of HLA expression or even an altered HLA ligandome displayed by leukemic cells via KIR binding (32–34).

Several limitations of the current study must be acknowledged. First, the conclusion is drawn from a retrospective analysis of a specific cohort. Generalizability to other ethnic groups or different haplo-HSCT platforms requires external validation. Second, the biological mechanisms remain incompletely defined. Future mechanistic studies should focus on the functional profiling of KIR3DL2^+^ NK cell subsets in the post-transplant period, their dynamic education, and their cytotoxic specificity against leukemia blasts in the context of haplo-HSCT. In particular, because there are significant differences in pathology and treatment between myeloid and lymphocid leukemia, their underlying mechanisms need to be explained separately. In conclusion, this large-scale analysis identifies the KIR3DL2-A3/A11 interaction as a key and previously underappreciated mediator of NK cell alloreactivity driven GVL in T cell-replete haplo-HSCT. Assessing KIR3DL2-A3/A11 interaction may offer a practical biomarker to optimize donor selection for relapse prevention in patients receiving Beijing protocol haplo-HSCT.

## Data Availability

The original contributions presented in the study are included in the article/supplementary material. Further inquiries can be directed to the corresponding authors.
